# miRNAs regulated overexpression of ryanodine receptor is involved in chlorantraniliprole resistance in *Plutella xylostella* (L.)

**DOI:** 10.1038/srep14095

**Published:** 2015-09-15

**Authors:** Xiuxia Li, Lei Guo, Xuguo Zhou, Xiwu Gao, Pei Liang

**Affiliations:** 1 Department of Entomology, China Agricultural University, Beijing, 100193, P. R. China; 2 College of Agronomy and Plant Protection, Qingdao Agricultural University, Qingdao, 266109, P. R. China; 3 Department of Entomology, University of Kentucky, Lexington, KY 40546-0091, USA

## Abstract

The amino acid mutations in ryanodine receptor (RyR) and elevated activity of detoxification enzymes have been associated with the diamide insecticide resistance in the diamondback moth, *Plutella xylostella* (L.). The up-regulation of *P. xylostella* RyR mRNA (*PxRyR*) expression has also been reported in field populations of different graphical origin. However, whether the up-regulation of *PxRyR* is involved in diamide resistance remains unknown. In this paper, 2.28- to 4.14-fold higher expression of *PxRyR* was detected in five field collected resistant populations, compared to that in a susceptible population. The expression of *PxRyR* was up-regulated 5.0- and 7.2-fold, respectively, after *P. xylostella* was treated with LC_50_ and LC_75_ of chlorantraniliprole for 12 h. Suppression of *PxRyR* using RNA interference restored the toxicity of chlorantraniliprole against the fourth instar larvae from the resistant population. More importantly, the expression of *PxRyR* is regulated by two miRNAs, miR-7a and miR-8519. These findings provide an empirical evidence of the involvement of miRNAs in the regulation of insecticide resistance, and shed light on the novel targets for the sustainable management of this devastating insect pest.

The diamondback moth, *Plutella xylostella* (L.), (Lepidoptera: Plutellidae), is a worldwide pest insect on cruciferous crops and causes $4–5 billions losses every year^1^. Chemical control with insecticides has been effective; however, *P*. *xylostella* has developed resistance to almost all classes of insecticides^2^,^3^, including chlorantraniliprole, a diamide insecticide used extensively to control lepidopteran pests^4^. The diamide insecticides have a novel mode of action by activating the ryanodine receptors (RyR) in muscle fibres and resulting in feeding cessation, muscle paralysis, and ultimately death^5^,^6^,^7^,^8^. High level of resistance to chlorantraniliprole (>2000-fold) has been reported in *P. xylostella* field populations in China, Thailand, Philippines and Brazil^8^,^9^,^10^.

The mechanistic study of chlorantraniliprole resistance suggests both quantitative and qualitative modifications in the resistant *P. xylostella*. The increased activity of detoxification enzymes such as cytochrome P450 monooxygenase (P450), carboxylesterase (CarE) and glutathione *S*-transferase (GSTs) might contribute to chlorantraniliprole resistance^11^,^12^. In addition a single point mutation (G4946E) in *PxRyR* was linked to the high level of resistance in the field populations collected from Philippines, Thailand, and China^4^,^10^. Most recently, combinations of four point mutations in *PxRyR* were demonstrated to play important roles in chlorantraniliprole resistance in a population collected in Yunnan province, China^13^. Besides mutation, overexpression of *PxRyR* mRNA has also been found in chlorantraniliprole resistant populations^14^,^15^. However, whether the overexpression of *PxRyR* is indeed involved in chlorantraniliprole resistance remains unclear.

MicroRNAs (miRNAs), a group of non-coding RNAs, 19–24 nt in length, modulate many biological processes, including development, metabolism, behavior and metamorphosis^16^,^17^,^18^, through post-transcriptional regulation either by degrading mRNA or blocking its translation^19^,^20^. As the target of diacylhydrazines insecticides, the expression of ecodysone receptor (EcR) is regulated by miRNA-281 in the silkworm, *Bombyx mori*^21^. Although miRNAs have been surveyed in *P. xylostella*^22^,^23^, none of them have been associated with the regulation of *PxRyR* expression.

In the present study, we demonstrated that the overexpression of *PxRyR* mRNA is involved in chlorantraniliprole resistance in *P. xylostella*. More importantly, the expression of *PxRyR* is regulated by two miRNAs, miR-7a and miR-8519. These findings provide an empirical evidence of the involvement of miRNAs in the regulation of insecticide resistance, and shed light on the novel targets for the sustainable management of this devastating insect pest.

## Results

### *PxRyR* expression in *P. xylostella* resistant populations

**Figure 1:**
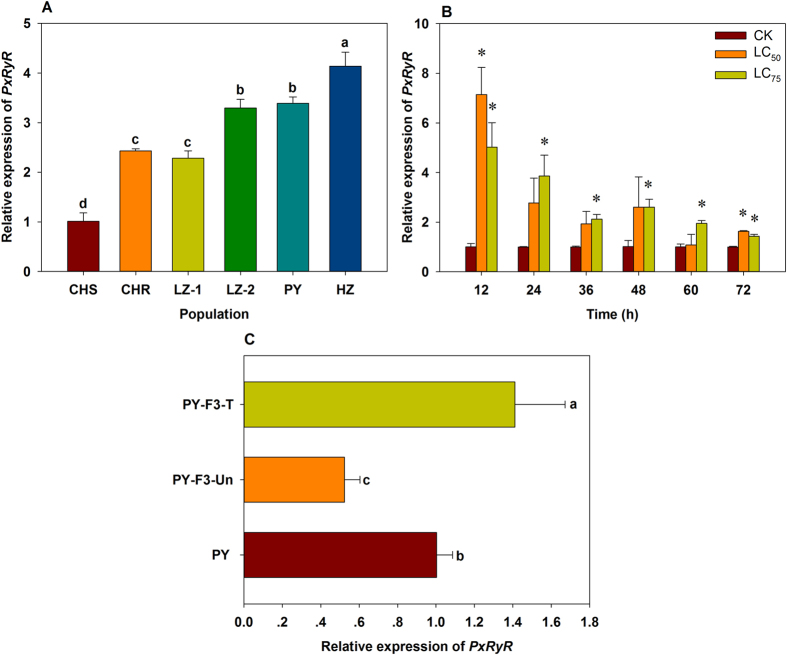
Relative expression of *PxRyR* in *Plutella xylostella*. (**A**) *PxRyR* expression in five resistant populations and a susceptible one. The relative expression of *PxRyR* in other five resistant populations was normalized to that in CHS population. (**B**) *PxRyR* expression in CHS population after treatment with LC_50_ or LC_75_ of chlorantraniliprole for different times. (**C**) *PxRyR* expression in different groups of PY population. Data presented as the mean ± SD for three independent replicates. The bars with different small letters in (**A**) and (**C**) are significantly different according to the one-way ANOVA, followed by Tukey’s multiple comparison test (*P* < 0.05), and the asterisks* in (**B**) represent the significant difference between the treatment and corresponding untreated control (Student’s *t*-test, *P* < 0.05).

*PxRyR* expression in four resistant field populations (HZ, PY, LZ-2 and LZ-1), a laboratory selected population (CHR) and a susceptible population (CHS) was compared using qRT-PCR. In comparison to CHS, *PxRyR* expressions in HZ, PY, LZ-2, LZ-1 and CHR were 4.14-, 3.39-, 3.30-, 2.28-and 2.43-fold higher, respectively (Fig. 1A). After treatment with LC_50_ and LC_75_ of chlorantraniliprole for 12 h, the expression of *PxRyR* in the susceptible CHS population increased 7.15- and 5.03-fold, respectively, than that of the untreated control (Fig. 1B). Similarly, expression of *PxRyR* was increased by 41% when the resistant PY population was selected with chlorantraniliprole continuously for two generations (PY-F3-T, Fig. 1C). In contrast, without exposure to any insecticide for two consecutive generations, *PxRyR* expression decreased by 48% (PY-F3-Un, Fig. 1C).

### *PxRyR* knockdown restored the susceptibility of resistant *P. xylostella* to chlorantraniliprole

**Figure 2:**
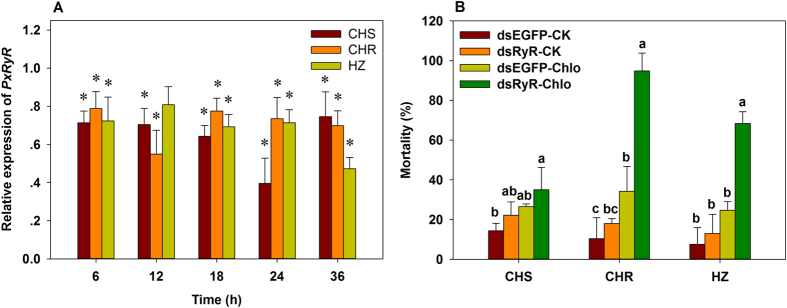
Relative expression of *PxRyR* in three populations of *P. xylostella* after injection of dsRyR for different time with injection of dsEGFP as control. The data was expressed as the ratio of treatment to the corresponding control (**A**), and the 96 h mortalities in susceptible and resistant populations caused by LC_25_ of chlorantraniliprole after injection of dsEGFP and dsRyR, the control group (CK) was treated with 0.1% Triton X-100 H_2_O. (**B**). Data were presented as the mean ± SD for three independent replicates. The asterisks*in (**A**) represent the significant difference between the treatment and the corresponding control by Student’s *t*-test (*P* < 0.05), and the bars with different small letters at same time point in (**B**) are statistical different (*P* < 0.05), tested by one-way ANOVA and followed by Tukey’s multiple comparison.

At 36 h post-injection of *PxRyR* dsRNA, the transcript levels of *PxRyR* in the third instar larvae from CHS, CHR and HZ populations decreased significantly by 25.4–60.5%, 21.2–45.1% and 19.2–52.8%, respectively at different time points compared with that of the control (Injection of dsEGFP)(Fig. 2A).

When exposed to LC_25_ of chlorantraniliprole for 96 h, no significant cumulative mortality difference were observed between *PxRyR* knockout and EGFP control groups in CHS, CHR and HZ populations (Fig. 2B). However, after treatment with LC_25_ of chlorantraniliprole, the *PxRyR* knockout groups showed a dramatic increase of morality by 32.0%, 169.6% and 135.2%, respectively, in all three populations (Fig. 2B), suggesting that RNAi-mediated knockdown of PxRyR mRNA expression restores the susceptibility of *P. xylostella* larvae to chlorantraniliprole.

### miR-7a and miR-8519 putatively regulate the expression of *PxRyR*

**Figure 3:**
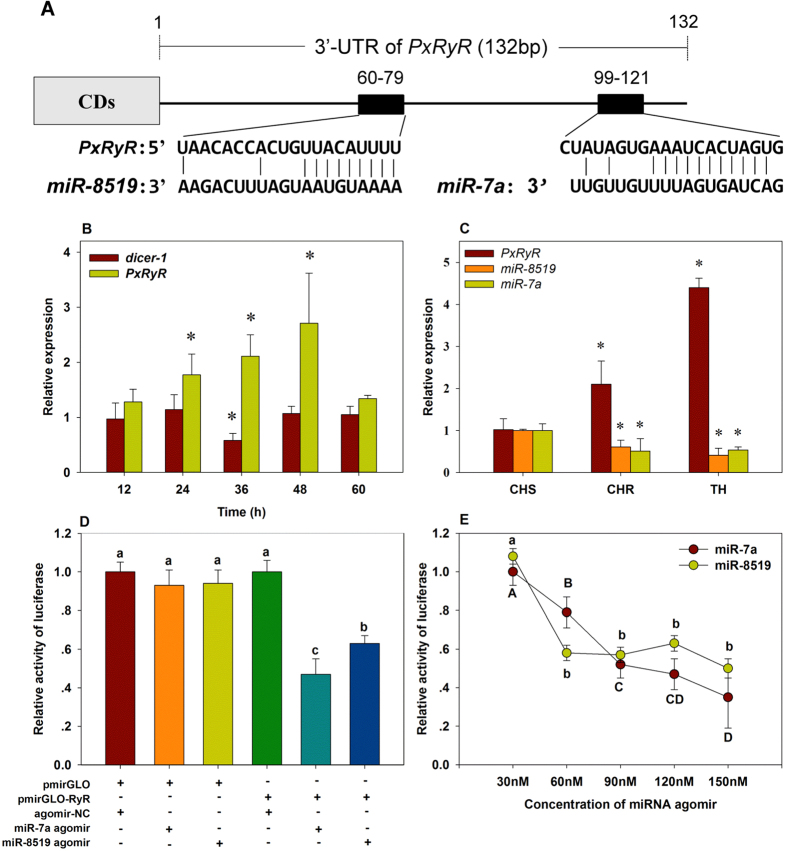
(**A**) Predicted target sites of miR-7a and miR-8519 in the 3′-UTR of *PxRyR*. (**B**) Relative expression of *PxRyR* after knockdown of *dicer-1*. (**C**) Relative expression of miR-7a, miR-8519 and *PxRyR* in CHS, CHR and TH populations. (**D**) Luciferase reporter assays performed by co-transfection of miR-7a or miR-8519 mimics with a luciferase reporter gene linked to the 3′-UTR of *PxRyR*. (**E**) Relative activity of firefly luciferase negatively co-related with the concentration of transfected miRNA mimics. All data are presented as the mean ± SD of three independent replicates. The bars marked with an asterisk * in (**B**,**C**) are significantly different (Student’s *t*-test, *P* < 0.05) with their corresponding control. In both (**D**,**E**), the firefly luciferase activity was normalized to Renilla luciferase activity and then normalized to the activity of the control group. Bars in (**D**) with different letters are statistically different, and in (**E**), small letters stand for significant difference for miR-8519, and the capital ones for miR-7a. (One-way ANOVA followed by Tukey’s multiple comparison tests, *P* < 0.05).

Pxy-miR-7a (miR-7a) and Pxy-miR-8519 (miR-8519) predicted bioinformatically can regulate the expression of *PxRyR*. In the 3′UTR of *PxRyR*, there is one potential binding site for miR-7a and miR-8519, respectively, with high complementarity (Fig. 3A). Specifically, the seed binding sites for miR-8519 and miR-7a are located at bases 72 to 78 and 114 to 120 of the 3′UTR, respectively (Fig. 3A).

To understand whether the expression of *PxRyR* was regulated by miRNAs, *dicer-1* was silenced by RNAi. The *dicer-1* expression decreased significantly at 36h (42%) post-injection of *dicer-1* dsRNA (Fig. 3B). In the meantime, the expression of *PxRyR* increased significantly (111%, Fig. 3B).

qRT-PCR results showed that the transcriptional level of miR-7a and miR-8519 in the resistant CHR and TH populations were significantly lower than that in the susceptible CHS population (Fig. 3C), while the expression level of their target gene *PxRyR* in CHR and TH were 2.1- and 4.4-fold higher than that in CHS population, respectively.

To determine whether or not miR-7a and miR-8519 could suppress *PxRyR* expression, the 3′-UTR of *PxRyR* was inserted into a pmirGLO vector to yield a recombined vector, pmirGLO-RyR. The firefly luciferase activity normalized to Renilla was significantly reduced after pmirGLO-RyR was co-transfected with agomir of miR-7a (Fig. 3D). A similar result was observed when pmirGLO-RyR was co-transfected with the agomir of miR-8519 (Fig. 3D). Moreover, the relative activity of firefly luciferase was negatively correlated with the concentration of miRNA agomir, especially for miR-7a (Fig. 3E). These results indicate that miR-8519 and miR-7a could regulate the expression of *PxRyR* by binding to its 3′-UTR.

### Expression of miR-7a or miR-8519 can modulate the susceptibility of *P. xylostella* to chlorantriliniprole

**Figure 4:**
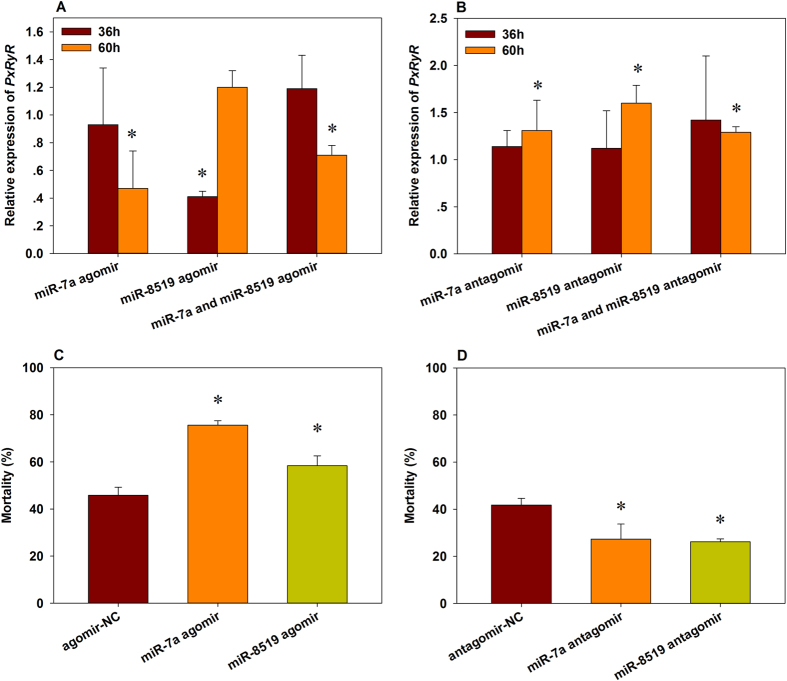
Relative expression of *PxRyR* (**A,B**) and mortalities (**C,D**) in CHS population of *Plutella xylostella* exposed to LC_50_ of chlorantraniliprole after injection of agomir or antagomir of miR-7a and miR-8519. Data presented as the mean ± SD for three independent replicates and the bars with asterisk* are significantly different with the corresponding control (Student’s *t*-test, *P* < 0.05).

To validate the expression of *PxRyR* is regulated by miR-7a and miR-8519, we injected the agomir (mimic) or antagomir (inhibitor) of miR-7a into the third instar larvae of *P. xylostella* and then examined the expressions of *PxRyR* at 36 and 60 h, respectively. Compared with the corresponding control group, the expression of *PxRyR* in miR-7a mimics injection group decreased by 53% (Fig. 4A) while increased by 31% (Fig. 4B) in miR-7a inhibitor injection group at 60 h post-treatment, respectively. And the expression of *PxRyR* also increased 60% at 60 h after the injection of miR-8519 inhibitor and decreased 59% at 36 h after the injection of miR-8519 mimic. As expected, increased expression (29%) was detected after the injection of a mixture of miR-7a and miR-8519 inhibitors (1:1 ratio), while the decreased expression (29%) was observed when a mixture of miRNA mimics was injected.

Mortality of *P. xylostella* larvae exposed to LC_50_ of chlorantraniliprole dramatically increased by 65.1% and 27.5% in miR-7a and miR-8519 mimics injection groups, respectively, in comparison to the mimic-NC injected control (Fig. 4C). While in the miR-7a and miR-8519 inhibitor injection groups, the larval mortality was significantly reduced by 34.7% and 37.3%, respectively (Fig. 4D). These results demonstrated that the fluctuation of miR-7a and miR-8519 expression can impact the susceptibility of *P. xylostella* to chlorantraniliprole through regulating the expression of *PxRyR*.

## Discussion

Mutations affecting the target protein and metabolism of insecticide are the most commonly described mechanisms conferring insecticide resistance^24^. In addition, the changed expression level of target proteins is also involved in insecticide resistance. The reduced expression of nicotinic acetylcholine receptor (nAChR) subunit *α2* has been proved to be responsible for the imidacloprid resistance in housefly, *Musca domestica*^25^. Target mutations and metabolism mediated insecticide resistance have been extensively studied in diamide insecticides resistant in *P. xylostella*^4^,^10^,^11^,^12^,^13^,^26^, while whether the changes of *PxRyR* expression is involved in resistance remains poorly understood. By comparing the tag-based digital gene expression (DGE) data, Lin *et al.*^26^ demonstrated that the expression of ryanodine receptor as well as other genes involved in calcium signaling pathways and muscle control pathways were down-regulated with the increase of chlorantraniliprole resistance in *P. xylostella* populations. However, Sun *et al.*^14^ and Yan *et al.*^15^ reported that the *PxRyR* overexpressed in different chlorantraniliprole or flubendiamide resistant populations of *P. xylostella*, and the expression of *PxRyR* can be induced by ryanodine and all three registered diamide insecticides respectively. In this paper, we also found the expression of *PxRyR* was significantly higher in four field collected high resistant populations as well as in a laboratory selected resistant one compared to the susceptible population, and both the short- and long-term treatment with chlorantraniliprole could induce the increase of the *PxRyR* expression in different populations. Combing these results, we conclude that the overexpression of *PxRyR* is most likely involved in diamide insecticide resistance in *P. xylostella*, because the same phenomenon has been found in multiple populations representing several field and laboratory selected resistant populations.

Further, we found that the RNAi-mediated knockdown of *PxRyR* expression reduced the larval tolerance to chlorantraniliprole, suggesting that the overexpression of *PxRyR* was involved in chlorantraniliprole resistance. Yang *et al.*^27^, however, reported that the repression of RyR mRNA level by RNAi greatly decreased chlorantraniliprole-induced mortality in white-backed planthopper *Sogatella furcifera*, suggesting that the down-regulation of RyR expression was involved in chlorantraniliprole resistance. It is not surprising that different resistant mechanisms were employed by different insect pests to the same insecticide.

### The invlovement of miRNA in insecticide resistance

MiRNAs comprise a large family of ~21-nucleotide-long RNAs that play key roles in post-transcriptional regulation of gene expression from plants to animals to viruses^19^,^28^. Functional studies indicate that miRNAs participate in the regulation of almost every cellular process^29^. In recent years miRNAs have attracted great interest in the field of toxicology. Lema and Cunninghama reviewed that the expression of miRNAs in both animals and plants is affected by several known toxicants, and proposed miRNAs may play an important role in toxicology^30^. More recently, several studies have proved that in human beings, the expression of cytochrome P450s (CYPs), important enzymes in catalyzing the metabolism of xenobiotics, and several nuclear receptors such as pregnane X receptor (PXR), the aryl hydrocarbon receptor (AhR), and the constitutive androstane receptor (CAR) are regulated by miRNAs^31^. Derecka *et al.* found that the expression of 15 miRNAs was affected by treatment of low-dose of imdacloprid in honeybee, *Apis mellifera*^32^. In this study, we provided evidence that miRNAs, miR-7a and miR-8519, are involved in diamide insecticide resistance in *P. xylostella* through up-regulation of *PxRyR* expression. These findings shed light on the roles of miRNAs in regulating insecticide resistance.

Regulation of gene expression can occur at different levels, such as modification of DNA, regulation of transcription and post-transcription. Here we revealed that the expression of *PxRyR* is regulated by miRNAs at the post-transcription level, however, other factors such as long non-coding RNA (lncRNA) may also play a role. It is worth noting that the expression levels of miR-7a in both susceptible (CHS) and resistant (CHR, TH and HZ) populations were 183.6-fold and 160.8–435.4-fold higher than that of miR-8519, respectively (Table 1). These quantitative differences suggest that these two miRNAs might play different roles in the regulation of *PxRyR*.

Though we proved here that the overexpression of *PxRyR* is involved in chlorantraniliprole resistance in *P. xylostella*, the relationship between the *PxRyR* overexpression and chlorantraniliprole resistance remains to be elucidated. Ziviani *et al.* found that the administration of nicotine to mice up-regulates the levels of RyR2, and this upregulation “was driven by the transcription factor CREB, and caused a long-lasting reinforcement of Ca^2+^signalling via the process of Ca^2+^-induced Ca^2+^ release^33^. RyR2 up-regulation was itself required for long-term phosphorylation of CREB in a positive-feedback signalling loop”. Therefore we speculated that the administration of chlorantraniliprole shares a similar mechanism in up-regulation of *PxRyR*. Diamide insecticides are potent activator of the RyR which may release stored calcium from the sarco-/endoplasmic reticulum to lumen^5^. And calcium is a common second messenger that regulates transcription processes in cells^34^. So when the PxRyR is activated by chlorantaniliprole, release of plenty of Ca^2+^ from the endoplasmic reticulum elevates the cytosolic Ca^2+^ levels and provides the Ca^2+^ trigger signals for a large number of physiological processes including the up-regulation of PxRyR. Then the newly expressed PxRyR compensate for the functional loss of PxRyR that has been continuously activated by chlorantaniliprole. But we still have no idea about the relationship between the administration of chlorantaniliprole and regulation of expressions of two miRNAs that target the *PxRyR*.

## Conclusions

In this study, we provided evidence that the overexpression of *PxRyR* was involved in chlorantraniliprole resistance in *P. xylostella*, and we proved that the *PxRyR* expression was regulated by miRNAs, miR-7a and miR-8519, via binding to its 3′-UTR. Furthermore, overexpression and suppression of both miRNAs modulated the susceptibility of *P. xylostella* to chlorantraniliprole. This is the first report showing that miRNAs could be involved in insecticide resistance through the regulation of target gene expression.

## Materials and Methods

### Insects and cell line

The susceptible CHS population was maintained in our laboratory for more than 10 years without exposure to any insecticide. The chlorantraniliprole resistant CHR population was derived from the CHS population by continuous selection with LC_50_ of chlorantraniliprole for more than 50 generations. The five resistant populations were collected in Chinese cabbage field from 2011 to 2013. The TH population was collected from Tonghai city, Yunnan province and the other four resistant populations were collected from Huizhou city (HZ), Lianzhou city (LZ-1 and LZ-2) and Panyu city (PY), Guangdong province. Insects were maintained at 27 ± 1 °C and a RH of 40–60% on radish seedlings (*Raphanuss ativus* L.) with a photoperiod of 16:8 h (L: D). *Plutella xylostella* adults were provided with 10% (W/V) honey solution as food and allowed to oviposit on radish seedlings.

The mammalian HEK293T cell line was maintained at 37 °C under 5% CO_2_ in DMEM high glucose medium (Gibco) containing 10% fetal bovine serum (Gibco).

### Quantification of *PxRyR* expression uing qRT-PCR

*PxRyR* expression in chlorantraniliprole resistant and susceptible populations, including 5 field, 1 laboratory, PY-F3-Un (PY population without exposure to any insecticides for two generations) and PY-F3-T (PY population selected with LC_50_ of chlorantraniliprole for two generations), were determined by qRT-PCR. To investigate the short term effect of chlorantraniliprole on *PxRyR* expression, the third instar larvae of CHS population were treated with LC_50_ or LC_75_ of chlorantraniliprole, respectively, for 72 h. Cabbage (*Brassica oleracea* var. *capitata* L.) leaf discs were dipped into chlorantraniliprole solution containing 0.1% Triton X-100 for 10 s, air-dried, and then placed into a 9 cm Petri dishes. Leaf discs treated with distilled water containing 0.1% Triton X-100 used as controls. A group of 20 newly molted third instar larvae (24 h within molting) were introduced into the Petri dish. In total, approximately 200 larvae were treated for each concentration, in which 10 larvae were collected at 12, 24, 36, 48, 60 and 72 h post-treatment, and each of 10 larvae was pooled for RNA extraction and qRT-PCR analysis. Three biological replicates were performed.

Total RNA samples were isolated from the fourth instar larvae using TRIzol kit (Invitrogen, Carlsbad, CA) according to the manufacturer’s instructions. The first-strand cDNA of mRNA and mature miRNA were synthesized using PrimeScript™ RT reagent Kit with gDNA Eraser (Perfect Real Time) (Takara Biotechnology, Dalian, China) and miScript II RT kit (Qiagen) following manufacturer’s instructions, respectively. qRT-PCR analysis was carried out using Platinum® SYBR® Green qPCRSuperMix-UDG Kit (Invitrogen) and ABI 7500 Real Time PCR system (Applied Biosystems). qRT-PCR reactions for miRNA or mRNA were as follows: initial incubation at 50 °C for 2 min and then 95 °C for 2 min, followed by 40 cycles of 95 °C for 15 s and 60 °C for 30 s. When the cycling protocol finished, melting curves were obtained by increasing the temperature from 60 to 95 °C to check the specificity of the primers. Standard curves were obtained using a 10-fold serial dilution of pooled cDNAs. qRT-PCR analyses were performed in triplicate and normalized to an internal control, U6 snRNA and ribosomal protein L32 mRNA for miRNA and mRNA, respectively. qRT-PCR data were analyzed using the 2^−ΔΔCt^ method^35^. All primers used in this study were listed in Table 2.

### *In vivo* RNAi

#### Suppression of *PxRyR*

The expression of *PxRyR* in three populations (CHS, CHR and HZ) was suppressed through injection of *PxRyR* dsRNA into the third instar larvae. About 0.5 μg of dsRNA was injected into each larva using a microinjector (Nanoliter 2000 Injector, WPI Inc.). Control larvae were injected with 0.5 μg of double stranded Enhanced Green Fluorescent Protein (dsEGFP). Injected larvae were placed on cabbage leaf discs to recover and reared under laboratory conditions. A total of 10 injected larvae were randomly collected at 6, 12, 18, 24 and 36 h post-injection for the subsequent qRT-PCR analysis. Toxicity assay against chlorantraniliprole was carried out 3 h post-injection. The injected larvae were allowed to feed on LC_25_ of chlorantraniliprole treated cabbage leaf discs for four days and the mortality was recorded at 96 h post-treatment. Larvae that did not move when pushed gently with a brush were considered dead. Over 50 larvae were treated in each replicate and three technical replicates were set up for each of the three biological replications.

#### Suppression of *dicer-1*

Dicer-1 is a key RNA endonuclease III required for mature miRNA processing^36^. If a target gene is post-transcriptionally regulated by miRNA(s), the knockdown of *dicer-1* mRNA would decrease the level of miRNA(s), and subsequently increase the mRNA expression of the target gene. To understand whether the expression of *PxRyR* is regulated by miRNA or not, the expression of *dicer-1* was interfered by microinjection of dsRNA of *dicer-1* into third instar larvae of CHS population. *Plutella xylostella* larvae were injected with 0.5 μg of *dicer-1* dsRNA and the control larvae were injected with 0.5 μg of dsEGFP. A total of 10 injected larvae were randomly collected at 12, 24, 36, 48 and 60 h post-injection, and each of 10 larvae was pooled for RNA extraction and qRT-PCR analysis. Three biological replicates were performed.

### dsRNA synthesis

The fragments of genes *PxRyR* (GENBANK accession JF926693.1) and *dicer-1* (http://iae.fafu.edu.cn/DBM/seqView.php?cds=Px004356) were amplified by reverse transcription PCR (RT-PCR) using specific primers conjugated with 20 bases of the T7 RNA polymerase promoter (Table 2). The PCR products, 667 bp for *PxRyR* and 633 bp for *dicer-1* were used as templates for dsRNA synthesis using the MEGAscriptRNAi kit (Ambion, Inc.). After synthesis, the dsRNA was isopropanol precipitated, resuspended in nuclease-free water, quantified with NanoDrop 2000 (Thermo Scientific) and stored at −20 °C until use.

### Quantification of two miRNAs and *PxRyR* expression

To understand the relationship between miRNAs and chlorantraniliprole resistance, expressions of miR-7a, miR-8519 and putative target *PxRyR* were determined in a susceptible CHS population and resistant populations CHR and TH using qRT-PCR. Moreover, the relative expression of these two miRNAs were determined in CHS, CHR, HZ and TH populations as well as in different larval stages within HZ population. A total of 10 individuals were used as a biological replicate for total RNA extraction and qPCR, and three replicates were carried out.

### Dual luciferase reporter (DLR) assay

A luciferase reporter plasmid pmirGLO-RyR was constructed by GenePharm (Shanghai, China) through inserting the full length 3′ UTR of *PxRyR* between the firefly luciferase ORF and SV40 poly (A) on pmirGLO vector. The vector is based on Promega dual-luciferase technology, with firefly luciferase (*luc2*) as the primary reporter to monitor mRNA regulation and Renilla luciferase (*hRluc-neo*) as a control reporter for normalization. For luciferase assay, HEK293T cells were cultured in a 96-well plate and transfected with plasmids pmirGLO-RyR and miRNA agomir (a dsRNA formed with the miRNA and its complimentary sequence) of each miRNA or agomir-NC (negative control, designed based on a *Caenorhabditis elegans* miRNA with no similarity to insect miRNAs) using Calcium Phosphate Cell Transfection Kit (Beyotime, Nanjing, China) according to the manufacturer’s instruction. Each well contained 0.2 μg plasmid while final concentration of miRNA agomir (mimics) varying from 30 nM to 150 nM. Luciferase assays were performed by using the Dual-Glo® Luciferase Assay System (Promega) 24 h post-transfection. Normalized firefly luciferase activity (firefly luciferase activity/Renilla luciferase activity) was compared to that of the control pmirGLO Vector. The mean of the relative luciferase expression ratio (firefly luciferase/renilla luciferase) of the control was set to 1. For each transfection, luciferase activity was averaged from five replicates.

### Modulation of miRNAs and the subsequent impacts on chlorantraniliprole susceptibility

MiRNA targets were predicted by a service provider (LC Sciences). The antagomir and agomir of miR-7a and miR-8519 were designed and synthesized by GenePharm Co. Ltd (Shanghai, China). The miRNA antagomir is the antisense oligonucleotides of the miRNA with a chemical modification. The negative control (NC) was designed based on a *C. elegans* miRNA with no similarity to insect miRNAs.

The third instar larvae from CHS population were selected for injection. For each larva, 138 nL of 40 μM agomir or antagomir was injected and the control was injected with agomir-NC or antagomir-NC, respectively. The mixture of agomir or antagomir of miR-7a and miR-8519 (1:1 ratio) was also injected. At 36 and 60 h post-injection, each ten larvae were collected and total RNA was extracted from whole larvae using TRIzol kit. Expression levels of *PxRyR* were determined by using qRT-PCR. Parallel assays were performed after injection of agomir or antagomir to determine the sensitivity of the injected larvae to the chlorantranilprole by treating the larvae with LC_50_ of chlorantraniliprole, and the mortalities were recorded after 96 h. The experiments were performed in triplicates.

## Additional Information

**How to cite this article**: Li, X. *et al.* miRNAs regulated overexpression of ryanodine receptor is involved in chlorantraniliprole resistance in *Plutella xylostella* (L.). *Sci. Rep.* **5**, 14095; doi: 10.1038/srep14095 (2015).

## Figures and Tables

**Table 1:** Relative expression of miR-7a compared with that of miR-8519 in different populations and instars of *Plutella xylostella*.

**Population**	**Relative expression of miR-7a**	**Larvae of HZ**	**Relative expression of miR-7a**
CHS	183.6 ± 11.6 **	1^st^ instar	204.9 ± 14.1 **
CHR	435.4 ± 50.6 **	2^nd^ instar	171.6 ± 3.3 **
HZ	281.6 ± 19.9 **	3^rd^ instar	88.9 ± 8.34 **
TH	160.8 ± 27.3 **	4^th^ instar	241.0 ± 4.8 **

Note: Data are ratios of relative expression of miR-7a to that of miR-8519, and presented as the mean ± SD for three independent replicates. The asterisks** represent a significantly different expression of miR-7a and miR-8519 (Student’s t-test, *P* < 0.01).

**Table 2:** Primers used for qRT-PCR analysis and dsRNA synthesis.

**Gene**	**Forward primer (5′-3′)**	**Reverse primer (5′-3′)**
*PxRyR*	CGCCAACAAGATGAGTGAGA	CCCGGTGTCGATGTAGTCTT
*L32*	ATCCGCCATCAGTCCGACCG	GGCTGAACCGTAACCAATGTTG
*miR-7a*	TGCCGACTAGTGATTTTGTTGTT	GAATCGAGCACCAGTTACGC
*miR-8519*	CGGCGAAAATGTAATGATTTC	GAATCGAGCACCAGTTACGC
*U6*	CGCAAGGATGACACGCAA	GAATCGAGCACCAGTTACGC
*dsRyR*	taatacgactcactataggg-TGTGAATTTCTGCGAAGACG	taatacgactcactataggg-TCATCCCCACTATGCTCTCC
*dsDicer-1*	taatacgactcactataggg-AGATGGAACCTTTGTGGCAG	taatacgactcactataggg-CGGTTATGATCCATTTTGGG
*dsEGFP*	taatacgactcactataggg-CAGTGCTTCAGCCGCTAC	taatacgactcactataggg-GTTCACCTTGATGCCGTTC

Note: Small letters are the sequence of T7 promotor.
